# Comparing incisional hernia risk between single-port and multiport robot-assisted partial nephrectomy: a retrospective analysis

**DOI:** 10.1007/s11701-026-03168-0

**Published:** 2026-04-10

**Authors:** Fabio Maria Valenzi, Valerio Santarelli, Hakan Bahadir Haberal, Juan Ramón Torres Anguiano, Luca Alfredo Morgantini, Ruben Sauer Calvo, Arianna Biasatti, Paolo Pietro Suraci, Yazan Al Salhi, Antonio Carbone, Simone Crivellaro

**Affiliations:** 1 Department of Urology, University of Illinois at Chicago, Chicago, IL USA; 2 Urology Unit, Department of Medico-Surgical Sciences and Biotechnologies, Faculty of Pharmacy and Medicine, Sapienza University of Rome, Via Franco Faggiana 1668, Latina, 04100 Italy; 3 Department of Maternal-Infant and Urological Sciences, Sapienza University of Rome, Umberto I Hospital, Rome, Italy; 4 Department of Medicine, Surgery and Health Sciences, Urologic Clinic, University of Trieste, Trieste, Italy

**Keywords:** Incisional hernia, Singleport, Multiport, Robotic surgery, Partial nephrectomy, Kidney tumor

## Abstract

Incisional hernia (IH) is a postoperative complication. No studies have investigated IH rates after Single Port (SP) partial nephrectomy (RAPN). This study aims to evaluate IH incidence after SP RAPN and to compare factors influencing IH in SP versus Multiport (MP) RAPN. Patients undergoing MP and SP RAPN were retrospectively included. Patients with less than 6 months follow-up, those undergoing concomitant procedures, converted to open or radical nephrectomy or with prior IH, were excluded from the study. A total of 279 patients (MP: 158(56.6%); SP: 121(43.4%)) were collected. IH developed in 12 patients (MP: 7 (4.4%); SP: 5 (4.1%)). In MP group, IH cases were more frequently associated with previous abdominal surgery (6 (85.7) vs. 22 (14.6); *p* < 0.001), higher BMI (36.4 (12.6) vs. 30.4 (9.25); *p* = 0.048) and presented with larger tumor size (4.0 (0.9) vs. 3.1 (1.6); *p* = 0.007). MP IH patients had larger tumors than those in SP group (4.0(0.9) vs. 2.8(0.4); *p* = 0.006). Stratified multivariable logistic regression demonstrated that previous abdominal surgery remained significantly associated with IH in the MP group (Odds ratio: 2.36; 95%CI: 1.06, 3.66; *p* < 0.001), while BMI was independently associated with IH only in MP RAPN (Odds ratio: 1.18; 95% CI: 1.02, 1.49; *p* = 0.028). IH rates after SP RAPN were comparable to those observed after MP RAPN. Established risk factors appeared to have a stronger impact in MP RAPN, while a lower observed incidence of IH was noted in selected SP subgroups, including those undergoing a retroperitoneal approach.

**Supplementary Information:**

The online version contains supplementary material available at 10.1007/s11701-026-03168-0.

## Introduction

Partial nephrectomy (PN) is the standard surgical treatment of pT1 kidney lesions. According to the latest guidelines, PN may also be considered for pT2 cases when technically feasible and in specific situations, such as patients with bilateral kidney lesions, a solitary kidney, or chronic kidney diseases (CKD) [1].

With the advancement of surgical technology, minimally invasive approaches have been developed to reduce the invasiveness of open surgery. Although no differences in oncological outcomes have been described between open or minimally invasive techniques, the latter are associated with lower morbidity, including shorter hospital stays and reduced blood loss [2]. However, minimally invasive techniques are not without postoperative complications. One potential issue is incisional hernia (IH), which can develop at the trocar-site and, in severe cases, lead to bowel incarceration requiring surgery [3].

In a large cohort of patients undergoing kidney tumor surgery, Hermann and colleagues reported a cumulative IH risk of 4.5%, with a higher incidence in open procedures compared to minimally invasive approach. However, specific IH rates for partial nephrectomy were not detailed in their study [4]. The current literature includes only a few single-center retrospective studies analyzing the incidence of IH after robotic multiport partial nephrectomy (MP RAPN). One study reported a radiologic IH diagnosis rate of 26.6%, with 11.7% of cases requiring surgical repair [5]. Another study, which focused solely on retroperitoneal MP RAPN, found a radiological IH incidence of 8% [6].

The da Vinci Single Port (SP) Platform (Intuitive Surgical Inc., Sunnyvale, CA) has been largely utilized for different urological procedures. This system features small, flexible instruments inserted through a single, larger trocar, compared to those of the MP [7]. For this reason, it has been hypothesized that SP surgery may lead to higher IH incidence than MP procedures [8]. So far, the risk of IH following robot assisted SP procedures has been investigated only in radical prostatectomy. Two studies found that SP radical prostatectomy carries a low IH risk, though the incidence is higher for the transperitoneal approach compared to the MP transperitoneal surgery [9, 10]. However, to date, no studies evaluated the incidence of IH following SP RAPN.

This study aims to determine the incidence of IH after SP RAPN and to compare risk factors associated with IH between SP and MP RAPN.

## Materials and methods

Patients with kidney lesion who underwent RAPN at the University of Illinois at Chicago from January 2014 to December 2024 were retrospectively included. All procedures were performed by three fellowship-trained surgeons at a single institution. The number of procedures included in the study was 48/121 (MP/SP) for Surgeon 1, 62/0 for Surgeon 2, and 48/0 for Surgeon 3. According to published learning curve thresholds, all surgeons had surpassed the learning curve for robotic partial nephrectomy [11]. The choice between MP and SP RAPN was based on surgeon discretion and institutional availability, as the SP platform was introduced at our institution in 2018, rather than on predefined patient or tumor specific selection criteria. Data were retrospectively extracted from the hospital’s electronic medical records (Epic^®^).

Postoperative IH was defined as a palpable hernia on physical examination and radiologically confirmed as protrusion of visceral organs or adipose tissue through the fascial incision site of the RAPN surgery. Patients with a follow-up less than six months or without available postoperative CT/MRI were excluded. Also excluded were those converted to open surgery and/or radical nephrectomy, undergoing concomitant surgeries, or with pre-existing hernia near the RAPN sites.

The study was conducted according to the declaration of Helsinki and approved by the Institutional Review Board (IRB: 2020 − 1428). All patients provided informed consent for both the surgical procedures and the collection of clinical data for research purposes.

Collected preoperative characteristics included: age, gender, race, body mass index (BMI), previous abdominal surgery, history of hernia, Charlson comorbidity index (CCI), American Society of Anesthesiologists (ASA) score, substance abuse, smoking status, history of hypertension, diabetes mellitus (DM), chronic obstructive pulmonary diseases (COPD), atherosclerotic cardiovascular disease (ASCVD), CKD stage and kidney lesion clinical stage. Intraoperative data included: surgical procedure (MP vs. SP), surgical approach (retro vs. transperitoneal), access type, warm ischemia time (WIT), estimated blood loss (EBL), total operative time and intraoperative complications. The following postoperative data were collected: length of stay (LOS), postoperative complications according to the Clavien-Dindo classification, pathological tumor size, IH occurrence, including time of onset and need for surgical repair.

### Surgical procedure

For MP RAPN patients were positioned in the flank position for both transperitoneal and retroperitoneal approaches. For transperitoneal cases, after inducing 15 mmHg pneumoperitoneum via a Verres needle, 3 to 4 robotic trocars were placed in a linear configuration, along with two assistant trocars (one of 12 mm and one of 5 mm). For retroperitoneal MP approach a 1 cm was made between the 12th rib tip and the iliac crest. The lumbodorsal fascia was pierced bluntly, and space expanded with balloon. Subsequently, 2 to 3 additional robotic trocars and two assistant trocars (one of 12 mm and one of 5 mm) were placed.

For the transperitoneal SP RAPN, a 3 to 4 cm pararectal incision was made to reach the fascia and peritoneum. Retroperitoneal SP was performed with two different approaches: flank position (like MP) or low anterior access (LAA) as described by Pellegrino et al. [12]. In all SP cases, an assistant trocar was inserted through the same skin incision but via a different fascial incision through the access port. The procedure was then conducted following the technique described by Palacios et al. [13].

For both MP and SP, a peri renal drain was placed at surgeon’s discretion. Fascia was closed with 0-Vycril suture in a running or figure of eight fashion, also based on surgeon preference. In MP procedures, specimen extraction was performed through the 12-mm assistant port. Port-site enlargement was performed only when required to allow safe intact specimen retrieval, with the extent of enlargement dictated by the specimen dimensions, without leading to specimen fragmentation.

### Statistical analysis

R software (version 4.4.1; R Foundation for Statistical Computing, Vienna, Austria) was used for the statistical analysis. Continues variables were expressed as median with interquartile range (IQR) while categorical variables were expressed as frequencies and percentage. Comparison between continuous variables was performed using Mann-Whitney U test while categorical variables were compared with chi-squared test or Fisher’s exact test based on the numerosity of the samples. Univariate logistic regression model was utilized to evaluated different variables on the incidence of IH for both robotic techniques. A multivariable logistic regression analysis with interaction terms between robotic technique and various clinical factors was then performed to determine the overall association between patient/surgical variables and IH development. Variables included in the multivariable analysis were selected based on clinical relevance and the results of the univariate logistic regression model. A two-sided p-value < 0.05 was considered statistically significant.

As a sensitivity analysis, propensity score matching was performed using 1:1 nearest-neighbor matching (caliper 0.2 of the logit propensity score). The propensity score model included previous abdominal surgery, BMI, pathological tumor size, age at surgery and gender. Covariate balance was assessed using standardized mean differences, with values < 0.10 considered optimal and values < 0.15 considered acceptable.

## Results

Among 327 patients who underwent MP and SP RAPN, 48 were excluded (37 lost to follow-up or without available imaging, 5 had concomitant surgery, 3 had pre-existing hernias along the surgical incision site and 3 converted to radical nephrectomy or open surgery). Thus, 279 patients were analyzed: 158 (56.6%) MP RAPN and 121 (43.4%) SP RAPN. The median follow-up was 42 months.

Baseline characteristics (Table 1) were homogenously distributed between the MP and SP groups except for a higher number of previous abdominal surgeries in the SP group (47(38.8%) vs. 28(17.7); *p* < 0.001) and a higher median Renal score in the MP group (6(2) vs. 5(2); *p* < 0.001). However, clinical tumor size showed no significant difference between MP and SP (3.35(1.5) vs. 3.0(1.7), respectively; *p* = 0.242).

**Table 1:** Baseline characteristics between multiport and singleport population

	MP *n* = 158 (56.6%)	SP *n* = 121 (43.4%)	*P* value
**Gender**			0.972
Male	86 (54.4)	67 (55.4)	
Female	72 (45.6)	54 (44.6)	
**Side**			0.187
Right	87(55.1)	77 (63.6)	
Left	71 (44.9)	44 (36.3)	
**Race**			0.749
Caucasian	31(19.6)	27 (22.3)	
African American	67 (42.4)	54 (44.6)	
Asian	4 (2.5)	4 (3.3)	
Hispanic	49 (31.01)	29 (23.97)	
Others	7(4.4)	7 (5.8)	
**Smoking**			0.095
No	85 (53.8)	52 (42.9)	
Yes	73 (46.2)	69 (57.1)	
**Substance Abuse**			0.630
No	140 (88.6)	104 (85.9)	
Yes	18 (11.4)	17 (14.1)	
**Hypertension**			0.638
No	55(34.8)	38 (31.4)	
Yes	103 (65.2)	83 (68.6)	
**Hypercholesterolemia**			0.822
No	99 (62.7)	78 (64.5)	
Yes	59 (37.3)	43 (35.5)	
**Diabetes**			0.279
No	120 (75.9)	84 (69.4)	
Yes	38 (24.1)	37 (30.6)	
**Obesity**			0.843
No	74 (46.8)	59 (48.8)	
Yes	84 (53.2)	62 (51.2)	
**ASCVD**			0.812
No	129 (81.6)	101 (83.5)	
Yes	29 (18.4)	20 (16.5)	
**COPD**			0.645
No	145 (91.8)	107 (88.4)	
Yes	13 (8.2)	14 (11.6)	
**CKD stage**			0.339
*≤* 3	154 (97.5)	115 (95.1)	
> 4	4 (2.5)	6 (4.9)	
**Previous Abdominal Surgery**			**< 0.001**
No	130 (82.3)	74 (61.2)	
Yes	28 (17.7)	47 (38.8)	
**Preop Hernia**			0.114
No	152 (96.2)	110 (90.9)	
Yes	6 (3.8)	11(9.1)	
**cT**			0.701
1	154 (97.5)	119 (98.4)	
2	4 (2.5)	2(1.6)	
	**Median (IQR)**	**Median (IQR)**	
**Age**	57.5 (17)	60 (15)	0.079
**BMI**	30.6 (9.5)	30.1 (9.8)	0.865
**CCI**	3 (3)	3 (3)	0.157
**RENAL score**	6 (2)	5 (2)	**< 0.001**
**Clinical Tumor Size**	3.35 (1.5)	3.0 (1.7)	0.242

MP: multiport; SP: single port; ASCVD: atherosclerotic cardiovascular disease; COPD: chronic obstructive pulmonary diseases; CKD: chronic kidney diseases; IQR: interquartile range: BMI: body mass index; CCI: Charlson comorbidity index

Postoperatively, 12(4,3%) developed IH: 7 in the MP group (4,4%) and 5 in the SP group (4,1%) with no statistically significant differences between the two robotic approaches (*p* = 0.989). All MP IHs occurred at the 12 mm trocar site. Median time to IH development was 4(3) months for the MP group and 4(7) months for the SP group with no significant differences between them (*p* = 0.742). SP had more retroperitoneal access than MP (90 (74,4) vs. 40 (25.3), respectively; *p* < 0.001).

Intraoperative complications occurred in 3 patients in the MP group (1 ureteral injury, 1 colon mural injury and 1 renal parenchyma injury) and in 2 patients in the SP group (1 injury to the renal parenchyma and 1 persistent pneumoperitoneum) and were all managed intraoperatively without the need for additional procedures.

When comparing patients who developed IH to those who did not by robotic approach (Table 2), previous abdominal surgery was statistically more frequent in patients developing IH in the MP group (6 (85.7%) vs. 22 (14.6); *p* < 0.001). Off-clamp technique was statistically significant higher in MP IH cases (4 (57.1) vs. 21 (13.9); *p* = 0.013). Drainage use was also significantly higher in MP among patients with IH (7(100.0%) vs. 2 (40.0); *p* = 0.045). Overall, MP had significantly higher drainage use than MP (22 (18.2) vs. 145 (91.8); *p* < 0.001). While obesity rates did not differ, BMI was significantly higher in patients who developed IH within the MP group (36.4 (12.6) vs. 30.4 (9.25); *p* = 0.048). Pathological tumor size did not differ between the MP and the SP groups (3.2 (1.5) vs. 3 (1.8), respectively; *p* = 0.547) but was higher in MP IH cases than MP without IH (4.0 (0.9) vs. 3.1(1.6); *p* = 0.007) and this was also significantly higher compared to patients who developed IH in the SP group (4.0 (0.9) vs. 2.8 (0.4); *p* = 0.006).

**Table 2:** Intraoperative and postoperative characteristics between robotic technique and development of incisional hernia

Variable	MP (*n* = 158)			SP (*n* = 121)			*P* value within the Hernia group according to robotic procedure
	No IH (*n* = 151)	IH (*n* = 7)	P value	No IH (*n* = 116)	IH (*n* = 5)	P value	
**Gender**			0.789			0.064	0.205
Male	82 (45.7)	4 (57.1)		62 (53.4)	5 (100.0)		
Female	69 (54.3)	3 (42.9)		54 (46.6)	0 (0.0)		
**Side**			0.459			0.987	0.785
Right	82 (54.3)	5 (71.4)		74 (63.8)	3 (60.0)		
Left	69 (45.7)	2 (28.6)		42 (36.2)	2 (40.0)		
**Smoking**			0.250			0.389	0.869
No	83 (54.9)	2 (28.6)		51 (43.9)	1 (20.0)		
Yes	68 (45.1)	5 (71.4)		65 (56.1)	4 (80.0)		
**Hypertension**			0.238			0.6487	0.813
No	51 (33.8)	4 (57.1)		36 (31.1)	2 (40.0)		
Yes	100 (66.2)	3 (42.9)		80 (68.9)	3 (60.0)		
**Diabetes**			0.197			0.641	0.152
No	113 (74.8)	7 (100.0)		81 (69.8)	3 (60.0)		
Yes	38 (25.2)	0 (0.0)		35 (30.2)	2 (40.0)		
**Obesity**			0.122			0.675	0.222
No	73 (48.3)	1 (14.3)		56 (48.3)	3 (60.0)		
Yes	78 (51.7)	6 (85.7)		60 (51.7)	2 (40.0)		
**ASCVD**			0.945			0.953	0.899
No	123 (81.5)	6 (85.7)		97 (83.6)	4 (80.0)		
Yes	28 (18.5)	1 (14.3)		19 (16.4)	1 (20.0)		
**COPD**			0.104			0.754	0.469
No	140 (92.7)	5 (71.4)		102 (87.9)	5 (100.0)		
Yes	11 (7.3)	2 (28.6)		14 (12.1)	0 (0.0)		
**Chronic kidney disease stage**			0.972			0.899	-
*≤* 3	147 (97.4)	7 (100.0)		110 (94.8)	5 (100.0)		
> 4	4 (2.6)	0 (0.0)		6 (5.2)	0 (0.0)		
**Previous Abdominal Surgery**			**< 0.001**			0.992	0.222
No	129 (85.4)	1 (14.3)		71 (61.2)	3 (60.0)		
Yes	22 (14.6)	6 (85.7)		45 (38.8)	2 (40.0)		
**Preoperative Hernia**			0.875			0.762	-
No	145 (96.1)	7 (100.0)		105 (90.5)	5 (100.0)		
Yes	6 (3.9)	0 (0.0)		11 (9.5)	0 (0.0)		
**ASA score**			0.451			0.653	0.926
1	7 (4.6)	0		3 (2.6)	0 (0.0)		
2	95 (95.9)	3 (42.9)		39 (33.6)	2 (40.0)		
3	49 (32.5)	4 (57.1)		73 (62.9)	3 (60.0)		
4	0 (0.0)	0		1 (0.9)	0 (0.0)		
**Anticoagulation/** **Antiplatelet**			0.675			0.053	0.222
No	118 (78.1)	6 (85.7)		95 (81.9)	2 (40.0)		
Yes	33 (21.9)	1 (14.3)		21 (18.1)	3 (60.0)		
**Clamping strategy**			**0.013**			0.065	0.687
Off-clamp	21 (13.9)	4 (57.1)		9 (7.8)	2 (40.0)		
On-clamp	130 (86.1)	3 (42.9)		107 (92.2)	3 (60.0)		
**Approach**			0.905			0.106	0.824
Transperitoneal	113 (74.8)	5 (71.4)		28 (24.1)	3 (60.0)		
Retroperitoneal	38 (25.2)	2 (28.6)		88 (75.9)	2 (40.0)		
**Incision type**			0.905			0.214	0.735
Ventral	113 (74.8)	5 (71.4)		28 (24.1)	3 (60.0)		
Subcostal	38 (25.2)	2 (28.6)		37 (31.8)	1 (20.0)		
LAA	-	-		51 (43.9)	1 (20.0)		
**Drainage**			0.916			0.224	**0.045**
No	13 (8.6)	0 (0.0)		96 (82.8)	3 (60.0)		
Yes	138 (91.4)	7 (100.0)		20 (17.2)	2 (40.0)		
**Post-operative complications**			0.351			0.262	-
No	122 (80.8)	7 (100.0)		110 (94.8)	5 (100.0)		
Yes	29 (19.2)	0 (0.0)		6 (5.2)	0 (20.0)		
**C-D complications**			-			-	-
< 3a	25 (89.3)	0 (0.0)		4 (66.7)	0 (0.0)		
> 3b	4 (10.7)	0 (0.0)		2 (33.3)	0 (0.0)		
**pT**			0.743			0.242	0.894
1	146 (96.7)	6 (85.7)		114 (93.9)	5 (100.0)		
2	5 (3.3)	1 (14.3)		2(6.1)	0 (0.0)		
**IH repair**							0.293
No	-	3 (42.9)		-	4 (80.0)		
Yes	-	4 (57.1)		-	1 (20.0)		
**Fascia suture**			0.841			0.762	0.712
Interrupted stitch	106 (70.2)	5 (71.4)		83 (71.6)	4 (80.0)		
Running suture	45 (29.8)	2 (28.6)		33 (28.4)	1 (20.0)		
	**MEDIAN (IQR)**			**MEDIAN (IQR)**			
**Age**	58 (16.5)	57 (19.5)	0.344	61 (14.5)	51 (10)	0.386	0.807
**BMI**	30.4 (9.25)	36.4 (12.6)	**0.048**	30.1 (9.48)	28.2 (11.6)	0.845	0.432
**OP time**	198 (71.5)	189 (110)	0.956	175 (76)	181 (61)	0.927	0.986
**EBL**	100 (150)	200 (200)	0.106	50 (124)	50 (70)	0.885	0.119
**LOS**	2 (1)	2(0.5)	0.289	0 (1)	1(1)	0.465	0.059
**WIT**	20 (8)	16 (8.5)	0.749	20 (11)	15 (2.5)	0.129	0.643
**Pathological tumor size**	3.1(1.6)	4.0 (0.9)	**0.007**	3.2 (1.8)	2.8 (0.4)	0.233	**0.006**
**Time to IH**	-	4 (3)	-	-	4 (7)	-	0.742

MP: multiport; SP: singleport; C-D: Clavien-Dindo classification; IH: incisional hernia; ASCVD: atherosclerotic cardiovascular disease; COPD: chronic obstructive pulmonary disease; BMI: body mass index; EBL: estimated blood loss; LOS: length of stay; WIT: warm ischemia time

To assess IH risk factors, patients were grouped by IH status (Table 3). Previous abdominal surgery was significantly more common in IH patients (8 (66.7) vs. 67 (25.1); *p* = 0.004). Off-clamp technique was also significantly more frequent in patients with IH (6 (50.0) vs. 30 (11.2); *p* = 0.002).

**Table 3:** Patients characteristics based on the development of incisional hernia

Variable	No IH (*n* = 267)	IH (*n* = 12)	*P* value
**Robot**			0.936
MP	151 (56.6)	7 (58.3)	
SP	116 (43.4)	5 (41.7)	
**Gender**			0.236
Male	144 (53.9)	9 (75.0)	
Female	123 (46.1)	3 (25.0)	
**Side**			0.766
Right	156 (58.4)	8 (66.7)	
Left	111 (41.6)	4 (33.3)	
**Smoking**			0.175
No	134 (50.2)	3 (25.0)	
Yes	133 (49.8)	9 (75.0)	
**Hypertension**			0.223
No	87 (32.6)	6 (50.0)	
Yes	180 (67.4)	6 (50.0)	
**Diabetes**			0.524
No	194 (72.7)	10 (83.3)	
Yes	73 (27.3)	2 (16.7)	
**Obesity**			0.384
No	129 (48.3)	4 (33.3)	
Yes	138 (51.7)	8 (66.7)	
**ASCVD**			
No	242 (90.6)	10 (83.3)	
Yes	25 (9.4)	2 (16.7)	
**COPD**			0.327
No	242 (90.6)	10 (83.3)	
Yes	25 (9.4)	2 (16.7)	
**Previous Abdominal Surgery**			**0.004**
No	200 (74.9)	4 (33.3)	
Yes	67 (25.1)	8 (66.7)	
**ASA score**			0.732
1	10 (3.7)	0	
2	134 (50.2)	5 (41.7)	
3	122 (45.7)	7 (58.3)	
4	1 (0.4)	0	
**Anticoagulation/Antiplatelets**			0.280
No	213 (79.8)	8 (66.7)	
Yes	54 (20.2)	4 (33.3)	
**Clamping strategy**			**0.002**
Off-clamp	30 (11.2)	6 (50.0)	
On-clamp	237 (88.8)	6 (50.0)	
**Approach**			0.391
Transperitoneal	141 (52.8)	8 (66.7)	
Retroperitoneal	126 (47.2)	4 (33.3)	
**Incision type**			0.677
Ventral	141 (52.8)	8 (66.7)	
Subcostal	75 (28.1)	3 (25.0)	
LAA	51 (19.1)	1 (8.3)	
**Drainage**			0.372
No	109 (40.8)	3 (25.0)	
Yes	158 (59.2)	9 (75.0)	
**pT**			0.299
1	260 (97.4)	11(91.7)	
2	7 (2.6)	1 (8.3)	
**Fascia suture**			0.841
Interrupted stitch	189 (70.8)	9 (75.0)	
Running suture	78 (29.2)	3 (25.0)	
	**Median (IQR)**		
**Age**	59 (15.5)	54 (13.2)	0.219
**BMI**	30.3 (9.3)	34.5 (15.0)	0.102
**Operative time**	187 (76)	200 (108)	0.692
**EBL**	80 (150)	135 (162)	0.210
**LOS**	2 (1)	1.5 (1)	0.873
**WIT**	20 (9)	15.5 (4)	0.206
**Pathological tumor size**	3.2 (1.8)	3.5 (1.3)	0.221

IH: incisional hernia; MP: multiport; SP: singleport; ASCVD: atherosclerotic cardiovascular disease; COPD: chronic obstructive pulmonary disease; BMI: body mass index; EBL: estimated blood loss; LOS: length of stay; WIT: warm ischemia time

Univariate logistic regression model was created separately for MP and SP procedures to determine factors associated with IH development (Table 4). Previous abdominal surgery resulted significantly correlated in the MP group (Odds ratio: 2.49; 95% CI: 1.92, 3.73; *p* > 0.001) while this correlation was not observed in the SP group (Odds ratio: 1.22; 95% CI: 0.8, 2.99; *p* = 0.892). Conversely, transperitoneal approach resulted significantly correlated within the SP group (Odds ratio: 0.18, 95% CI: 0.02, 0.98; *p* = 0.047) but not in the MP group (Odds ratio: 1.34; 95% CI: 0.23, 5.82; *p* = 0.714). On-clamp technique showed significance only in the SP group (Odds ratio: 0.12; 95% CI: 0.02, 0.82; *p* = 0.032). Lastly, BMI was significant in the MP group (Odds ratio: 1.18; 95% CI: 1.01, 1.17; *p* = 0.025) but not in the SP group (Odds ratio: 1.04; 95% CI: 0.93, 1.14; *p* = 0.469).

**Table 4:** Univariate logistic regression analysis predicting IH based on robotic approach

Variable	MP OR (95% CI)	*P* value	SP OR (95% CI)	*P* value
**Smoking**	2.68 (0.62, 3.53)	0.188	2.36 (0.42, 3.41)	0.345
**Hypertension**	0.39 (0.09, 1.69)	0.208	0.64 (0.12, 3.95)	0.599
**Diabetes**	0.19 (0.01, 1.68)	0.164	1.64 (0.26, 4.81)	0.568
**ASCVD**	0.55 (0.13, 2.54)	0.422	1.26 (0.22, 2.85)	0.811
**COPD**	3.55 (0.92, 6.26)	0.060	0.64 (0.01, 6.18)	0.756
**Obesity**	2.06 (1.83, 3.96)	0.088	0.67 (0.11, 3.56)	0.631
**Previous Abdominal surgery**	2.49 (1.92, 3.73)	**< 0.001**	1.22 (0.18, 2.99)	0.892
**Approach**				
Transperitoneal	0.05 (0.02, 0.10)	-	0.15 (0.04, 0.40)	-
Retroperitoneal	1.34 (0.23, 5.82)	0.714	0.18 (0.02, 0.98)	**0.047**
**Clamping strategy**				
Off-clamp technique	0.16 (0.04, 0.45)	-	0.26 (0.05, 0.93)	-
On-clamp technique	0.21 (0.05, 1.01)	0.051	0.12 (0.02, 0.82)	**0.032**
**pT**				
1	0.08 (0.01,0.13)	-	0.61 (0.01, 0.99)	-
2	2.87 (0.29, 4.84)	0.179	0.02 (0.01, 0.99)	0.996
**Drainage**	0.146 (1.16, 1.93)	0.788	3.36 (0.53, 5.46)	0.181
**Fascia suture**	1.06 (0.19, 4.60)	0.937	0.83 (0.08, 4.71)	0.846
**Surgical incision type**				
Ventral	0.05 (0.02, 0.10)	-	0.12 (0.03, 0.33)	-
Subcostal	1.34 (0.23, 3.82)	0.714	0.33 (0.03, 2.11)	0.237
LAA	-	-	0.24 (0.02, 1.52)	0.130
**Age**	0.96 (0.91, 1.02)	0.230	0.98 (0.92, 1.05)	0.509
**EBL**	1.01 (1.00, 1.03)	0.111	1.00 (0.99, 1.01)	0.874
**Operative time**	1.01 (0.99, 1.06)	0.773	1.01 (0.98, 1.04)	0.586
**Pathological tumor size**	1.56 (0.93, 2.57)	0.091	0.71 (0.29, 1.32)	0.529
**BMI**	1.09 (1.01, 1.17)	**0.025**	1.04 (0.93, 1.14)	0.469

MP: multiport; SP: singleport; IH: incisional hernia; ASCVD: atherosclerotic cardiovascular disease; COPD: chronic obstructive pulmonary disease; BMI: body mass index; EBL: estimated blood loss; LOS: length of stay

**Fig. 1:**
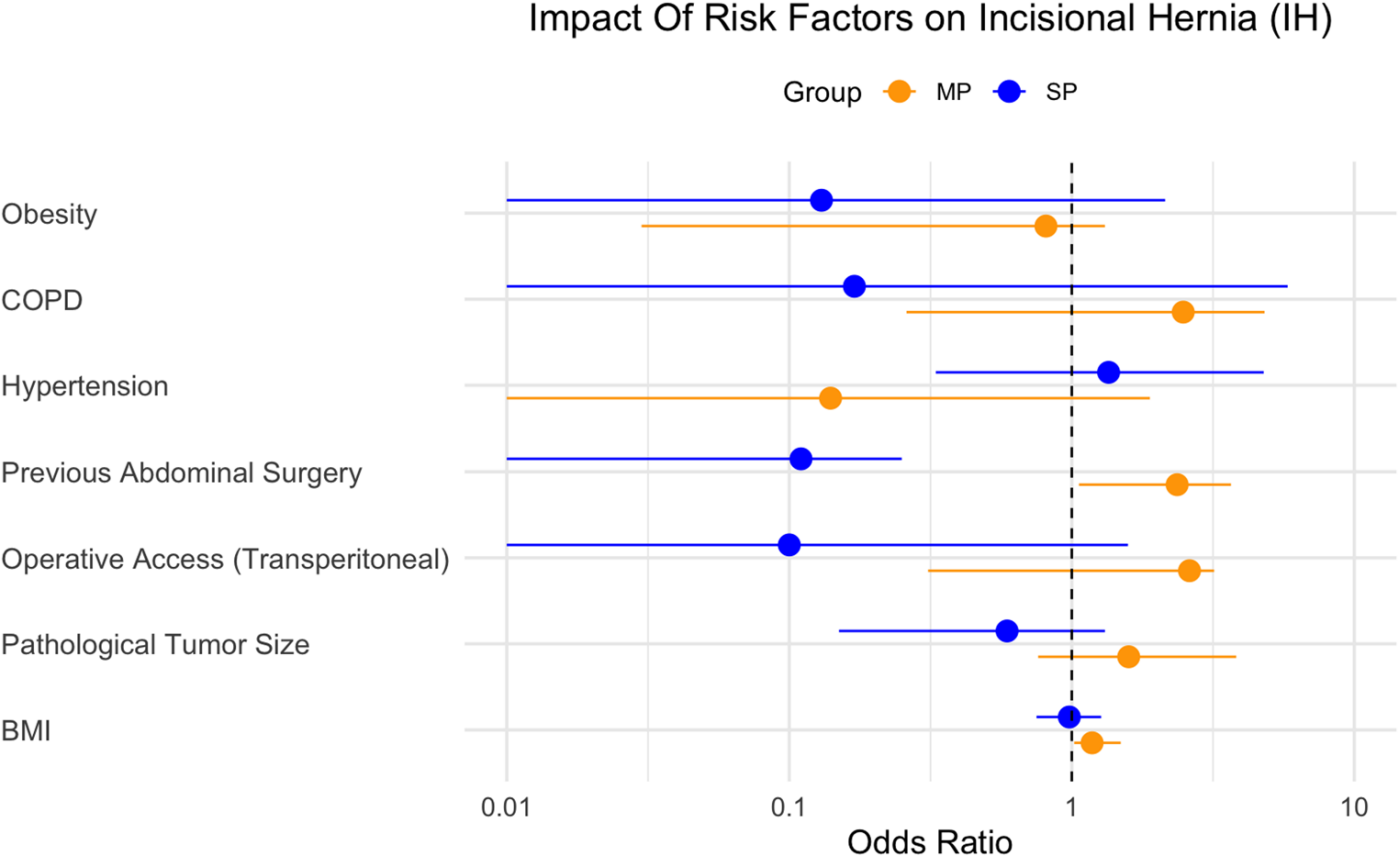
Forest Plot of risk factors for IH by surgical approach based on the stratified multivariable regression analysis. MP: multiport; SP: singleport; COPD: chronic pulmonary diseases; BMI: body mass index

In stratified multivariable logistic regression analysis (Table 5), previous abdominal surgery remained significant in the MP group (Odds ratio: 2.36; 95% CI: 1.06, 3.66); *p* < 0.001) and also became significant in the SP group (Odds ratio: 0.11; 95% CI: 0.01, 0.25; *p* = 0.003). BMI remained significant only in the MP group (Odds ratio: 1.18; 95% CI: 1.02, 1.49; *p* = 0.028) but not in the SP group (Odds ratio: 0.98; 95% CI: 0.75; 1.27; *p* = 0.885). Pathological tumor size, operative access, hypertension, COPD and obesity were not significantly associated with IH in either group (Fig. 1).

**Table 5:** Stratified multivariable logistic regression model with interaction between robotic technique and different indipendent variables predicting IH

Variable	MP OR (95% CI)	*P* value	SP OR (95% CI)	*P* value
Obesity	0.81 (0.03, 1.31)	0.892	0.13 (0.01, 2.14)	0.348
COPD	2.48 (0.26, 4.81)	0.417	0.17 (0.01, 5.81)	0.349
Hypertension	0.14 (0.01, 1.89)	0.156	1.35 (0.33, 4.78)	0.268
Previous Abdominal Surgery	2.36 (1.06, 3.66)	**< 0.001**	0.11 (0.01, 0.25)	**0.003**
Operative access (Transperitoneal)	2.61 (0.31, 3.19)	0.376	0.10 (0.01, 1.58)	0.094
Pathological Tumor Size	1.59 (0.76, 3.82)	0.208	0.59 (0.15, 1.31)	0.193
BMI	1.18 (1.02, 1.49)	**0.028**	0.98 (0.75, 1.27)	0.885
Intercept	0.01 (0.00, 0.12)	< 0.001	0.01 (0.00, 0.21)	0.001

MP: multiport; SP: singleport; COPD: chronic pulmonary diseases; BMI: body mass index

As a sensitivity analysis, propensity score matching was performed to address baseline imbalances between the two groups. After matching, 99 patients were included in each group with substantially improved covariate balance (all standardized mean differences < 0.15), including complete balance for previous abdominal surgery (supplementary Table 1). In the matched cohort, incisional hernia occurred in 6/99 patients (6.1%) in the multiport group and in 4/99 patients (4.0%) in the single-port group, with no statistically significant difference between groups (OR 0.65, 95% CI 0.13–2.86; *p* = 0.75).

## Discussion

Advancements in surgical technology have continually aimed to improve procedural quality for both surgeons and patients. Minimally invasive surgery allows performing complex surgery through less invasive approach, resulting in smaller skin incisions [14]. However, even small incisions such as those used for trocar placement, can lead to complications, including IH [15]. With the introduction of the SP surgical platform, concerns have arisen regarding the potential impact that a larger incision compared to those of the MP or laparoscopic techniques might have in terms of IH development [16]. To date, this is the first study that compares the incidence of IH between MP and SP RAPN.

In this study, the overall incidence of IH after RAPN was 4.3% with similar rates between MP (4.4%) and SP procedures (4.1%). These findings align with the existing literature, which reports IH rates up to 5.2% following laparoscopic procedures and 4.5% after kidney surgery [4, 17]. The median time to IH development was also comparable between the two robotic groups, occurring at 4(3) months in the MP group and 4(7) months in the SP group. Despite the long follow-up (median: 42 months), IH cases predominantly emerged in the early postoperative months, with only one case developing more than a year after surgery. This pattern is also consistent with previous studies, which indicate that while IH can develop from the early postoperative period to several months later, the majority appear shortly after surgery [5].

Several studies have identified risk factors for IH including higher BMI, previous abdominal surgery, tumor size, smoking habits, hypertension, COPD, ASCVD and end-stage chronic kidney diseases [6, 18, 19].

In our study, a history of previous abdominal surgery was significantly more frequent among patients who developed IH, with this association being particularly evident in the MP group. Multivariable logistic regression analysis further revealed that previous abdominal surgery was associated with an increased risk of IH in the MP cohort. In contrast, within the SP cohort, a lower observed incidence of IH was noted among patients with previous abdominal surgery. However, this finding should be interpreted cautiously given the limited number of events. This finding is particularly noteworthy, given that SP patients had a higher baseline prevalence of previous abdominal surgery compared to the MP group.

Conversely, BMI resulted significantly higher among patients who developed IH in the MP group compared to those in the SP group, despite no significant baseline differences. Furthermore, multivariable regression analysis demonstrated that each unit increase in BMI was associated with an 18% higher risk of IH in the MP group. This supports findings from prior studies, such as the HERNIA Project, which identified BMI *≥* 25 as a risk factor for IH [18]. However, no significant correlation between BMI and IH was observed in the SP group, although the likelihood ratio suggested that SP surgery may mitigate the impact of BMI on IH development.

Pathological tumor size was another significant factor in IH development within the MP group, with patients who developed IH having significantly larger tumors that those in the SP IH group. However, multivariable analysis did not confirm this correlation thus suggesting higher likelihood for developing IH in the MP than SP, hypothesizing a protective role of SP concerning this feature. This would align with the findings of Ghoreifi et al., who reported an association between pathological tumor stage *≥* 2 with IH following robotic nephrectomy [5]. In our study, pathological stage itself was not statistically significant nor correlated with IH in both robotic groups, likely due to the small sample size, with only eight patients classified as pT2. A possible explanation for the observed differences between MP and SP procedures lies in the incision technique. Unlike MP procedures, which might require enlargement of the 12 mm assistant trocar site for specimen extraction, SP procedures involve a predetermined 3–4 cm incision from the outset. This carries a clearer exposition of the fascia, whereas in MP procedures, trocar site enlargement is often performed without optimal fascial exposure. These factors may contribute to explain the discrepancies in IH risk between MP and SP techniques regarding tumor size.

Regarding the surgical approach, no significant differences were found between transperitoneal and retroperitoneal access in relation to IH development within each robotic group. However, univariate analysis showed that SP patients undergoing a retroperitoneal approach had an 82% lower likelihood of developing IH compared to those undergoing a transperitoneal approach. Although, this association did not reach statistical significance in the multivariable analysis, the odds ratio trend favored SP, suggesting a possible protective effect of retroperitoneal access in SP procedures. On the contrary, the absence of significant differences in the MP are in line with several studies, which showed no significant differences in postoperative complication, including IH, between transperitoneal and retroperitoneal partial nephrectomy [5, 20]. When examining IH incidence specifically within the SP group, retroperitoneal cases had a lower rate (2/88, 2.27%) compared to transperitoneal cases (3/28, 10.7%). Similarly, different incision types were associated with varying IH rates, with the lowest incidence observed in the low anterior access (LAA) approach (1/51, 1.9%), followed by the flank position (1/37, 2.7%), and the highest rate in ventral (transperitoneal) incisions (3/28, 10.7%). Although not statistically significant, these trends suggest that retroperitoneal access, particularly the LAA, may be associated with a lower risk of IH when performing SP RAPN. This aligns with the findings of Lambertini et al. who reported lower complication rates and reduced morbidity with retroperitoneal SP RAPN compared to MP [21]. Additionally, retroperitoneal access in SP surgery has already been shown to reduce postoperative complications rates, particularly in frail and elderly patients who are at increased risk of IH [22–24].

Interestingly, clamping strategy was also significantly different between IH and non-IH groups, with higher prevalence of off-clamp procedures among patients who developed IH, particularly in MP IH cases compared to MP non-IH cases. Univariate analysis, revealed that on-clamp technique was significantly associated with a lower risk of IH in the SP group, suggesting a potential protective role over the off-clamp technique. However, this finding should be interpreted cautiously due to the small sample size and the imbalance in the distribution of on-clamp versus off-clamp procedures. Moreover, studies are still controversial regarding differences in postoperative complication rates between on-clamp and off-clamp RAPN, and no studies have specifically examined the correlation between clamping strategy and IH following PN [25, 26].

Regarding fascial closure, the European Hernia Society (EHS) recommends continuous, single-layer, non-rapidly absorbable sutures for optimal closure [27]. In our cohort, all cases utilized Vicryl sutures, with either a running or uninterrupted technique. While fascial closure is critical in IH prevention, especially in incisions larger than 10 mm and those used for specimen extraction [28], our small sample size was insufficient to establish a significant correlation between closure technique and IH development.

This study has several limitations. Although incision size is a well-recognized risk factor for IH, the final length of the extraction incision in MP procedures could not be quantitatively analyzed due to the retrospective nature of the study and the lack of systematic documentation in operative reports. Specimen extraction in MP procedures was specimen-driven and performed through the 12-mm assistant port, which was enlarged only when necessary to allow intact retrieval. In this context, pathological tumor size, directly influencing the need for incision enlargement, was included in the analysis and was significantly associated with IH in the MP cohort, supporting its role as a clinically meaningful surrogate. To further account for the potential confounding effect of tumor size and other baseline imbalances inherent to the retrospective design, a propensity score–matched sensitivity analysis was performed, yielding results consistent with the primary analysis. Standardized prospective documentation of final incision length would allow a more accurate assessment of its role in incisional hernia development.

In addition, the low number of IH events limits the reliability of multivariable analyses overall, particularly in subgroup analyses, and increases the risk of overfitting and unstable estimates.

Moreover, some technical variables known to influence incisional hernia risk such as, extraction-site characteristics and fascial closure technique, were not fully standardized and could not be robustly analyzed due to the low number of IH events, potentially contributing to residual technical heterogeneity.

Furthermore, this was a retrospective, single-center study involving procedures performed by three different surgeons, which may introduce some degree of inter-operator variability. Although standardized operative protocols were adopted, such variability cannot be completely excluded. As a single-center experience involving fellowship-trained surgeons with established expertise in robotic PN, the generalizability of these findings to lower-volume centers or surgeons earlier in their learning curve may be limited.

Moreover, the non-randomized nature of the study and the absence of predefined selection criteria for MP versus SP RAPN raise the possibility of residual confounding related to patient selection, tumor characteristics, anatomy, and surgeon-related factors that could not be fully accounted for, despite multivariable adjustment and propensity score matching.

For this reason, further studies with larger cohorts are warranted to better evaluate the factors influencing IH development after SP RAPN and to further compare these findings with MP RAPN.

### Conclusions

In conclusion, this study shows that MP and SP RAPN are associated with comparable rates of IH. While established risk factors appear to significantly influence IH occurrence following MP RAPN, their impact seems less pronounced in SP procedures. An association between SP RAPN and a lower observed incidence of IH was noted in patients with previous abdominal surgery, as well as in those with higher BMI and larger tumors. However, these findings should be interpreted cautiously and considered hypothesis-generating given the retrospective observational design and the limited number of events. Furthermore, within the SP cohort, retroperitoneal access may be associated with a lower IH rate.

## Supplementary Information

Below is the link to the electronic supplementary material.

Supplementary Material 1

## Data Availability

The data supporting the findings of this study were obtained from the institutional electronic medical record system (EPIC). The data are not publicly available due to privacy and ethical restrictions involving patient information. De-identified data may be made available from the corresponding author upon reasonable request, subject to institutional approval.

## Abbreviations

PN: Partial Nephrectomy
IH: Incisional Hernia
SP: Single Port
MP: Multiport
RAPN: Robot-assisted Partial Nephrectomy
BMI: Body Mass Index
CCI: Charlson Comorbidity Index
ASA: American Society of Anesthesiologists score
DM: Diabetes Mellitus
COPD: Chronic Obstructive Pulmonary Diseases
ASCVD: Atherosclerotic Cardiovascular Disease
CKD: Chronic Kidney Disease stage
WIT: Warm Ischemia Time
EBL: Estimated Blood Loss
LOS: Length of Stay
LAA: Low Anterior Access
IQR: Interquartile Range
EHS: European Hernia Society
