# Supplementary material for: Comparing incisional hernia risk between single-port and multiport robot-assisted partial nephrectomy: a retrospective analysis
Source: J Robot Surg. 2026 Apr 10;20(1):425. doi: 10.1007/s11701-026-03168-0 (PMC13068765; doi:10.1007/s11701-026-03168-0)
Supplement: Supplementary file 1 — Supplementary Material 1 [file 11701_2026_3168_MOESM1_ESM.docx]

| **Variable** | **Unmatched MP** | **Unmatched SP** | **SMD (Before)** | **Matched MP** | **Matched SP** | **SMD (After)** |
| --- | --- | --- | --- | --- | --- | --- |
| **Previous Abdominal surgery, n (%)** | 28 (17.7) | 47 (38.8) | 0.43 | 25 (25.3) | 25 (25.3) | 0.00 |
| **BMI, median (IQR)** | 30.6 (9.5) | 30.1 (9.8) | 0.04 | 30.8 (9.2) | 30.0 (9.6) | 0.04 |
| **Pathological Tumor Size, median (IQR)** | 3.2 (1.5) | 3.0 (1.8) | 0.11 | 3.2 (1.6) | 3.3 (1.7) | 0.01 |
| **Age, median (IQR)** | 57.5 (17) | 60 (15) | 0.15 | 59 (16.5) | 63 (15.5) | 0.14 |
| **Gender male, n (%)** | 86 (54.4) | 67 (55.4) | 0.02 | 63 (63.6) | 61 (61.6) | 0.04 |

**Supplementary Table1** Baseline characteristics before and after propensity score matching. MP: multiport; SP: singleport; SMD: Standardized mean differences; BMI: body mass index.
